# Testosterone protects from metabolic syndrome-associated lung dysfunction in a high-fat diet rabbit model

**DOI:** 10.1210/endocr/bqag048

**Published:** 2026-04-16

**Authors:** Giulia Guarnieri, Paolo Comeglio, Sandra Filippi, Ilaria Cellai, Gabriele Acciai, Gianluca Bartolucci, Alessandro Pini, Amedeo Amedei, Ludovico Silvestri, Rachele Garella, Maria Emanuela Ragosta, Sarah Cipriani, Sara Marchiani, Giulia Rastrelli, Mario Maggi, Annamaria Morelli, Linda Vignozzi

**Affiliations:** Department of Experimental and Clinical Medicine, University of Florence, 50139 Florence, Italy; Department of Experimental and Clinical Biomedical Sciences “Mario Serio”, University of Florence, 50139 Florence, Italy; Department of Neurosciences, Psychology, Drug Research and Child Health (NEUROFARBA), University of Florence, 50139 Florence, Italy; Department of Experimental and Clinical Biomedical Sciences “Mario Serio”, University of Florence, 50139 Florence, Italy; Department of Experimental and Clinical Biomedical Sciences “Mario Serio”, University of Florence, 50139 Florence, Italy; Department of Neurosciences, Psychology, Drug Research and Child Health (NEUROFARBA), University of Florence, 50139 Florence, Italy; Department of Experimental and Clinical Medicine, University of Florence, 50139 Florence, Italy; Department of Experimental and Clinical Medicine, University of Florence, 50139 Florence, Italy; European Laboratory for Non-Linear Spectroscopy (LENS), University of Florence, 50019 Sesto Fiorentino, Italy; Department of Physics and Astronomy, University of Florence, 50019 Sesto Fiorentino, Italy; Department of Experimental and Clinical Medicine, University of Florence, 50139 Florence, Italy; Department of Experimental and Clinical Biomedical Sciences “Mario Serio”, University of Florence, 50139 Florence, Italy; Department of Experimental and Clinical Biomedical Sciences “Mario Serio”, University of Florence, 50139 Florence, Italy; Department of Experimental and Clinical Biomedical Sciences “Mario Serio”, University of Florence, 50139 Florence, Italy; Department of Experimental and Clinical Biomedical Sciences “Mario Serio”, University of Florence, 50139 Florence, Italy; Department of Experimental and Clinical Biomedical Sciences “Mario Serio”, University of Florence, 50139 Florence, Italy; Interuniversity Consortium “Istituto Nazionale Biostrutture e Biosistemi” (INBB), 00136 Rome, Italy; Department of Experimental and Clinical Medicine, University of Florence, 50139 Florence, Italy; Department of Experimental and Clinical Biomedical Sciences “Mario Serio”, University of Florence, 50139 Florence, Italy; Interuniversity Consortium “Istituto Nazionale Biostrutture e Biosistemi” (INBB), 00136 Rome, Italy

**Keywords:** metabolic syndrome, high-fat diet, testosterone, lung, inflammation, fibrosis

## Abstract

Metabolic syndrome (MetS), including obesity, dyslipidemia, hypertension, insulin resistance, and often testosterone (T) deficiency, is increasingly linked to impaired lung function, worsened by systemic inflammation. COVID-19 highlighted the vulnerability of metabolically impaired patients to respiratory complications. Preclinical mechanistic studies remain limited. This study examined MetS effects on lung function and morphology, and the impact of T therapy in a high-fat diet (HFD)-induced MetS rabbit model. Male New Zealand White rabbits were assigned to: regular diet, HFD 6 weeks, HFD 12 weeks (HFD12W), HFD + T 12 weeks (HFD + T12W), and HFD + T last 6 weeks (HFD12W + T6W). Lung function was measured via airway opening pressure (PAO), and tissues analyzed for macrophages (RAM11), collagen (picrosirius red), and inflammatory/fibrotic gene expression. HFD induced MetS features, hypogonadism, increased PAO, reduced compliance, elevated fatty acids, and early macrophage remodeling. At 12 weeks, inflammation and fibrosis were prominent, with upregulation of *IL1β*, *LOX1*, *RORγt*, *TLR2*, *COL1A1*, *COL3A1*, and *TGFβ1*. T therapy increased plasma T, improved metabolic parameters, reduced PAO, and reversed inflammatory/fibrotic gene expression. Histology confirmed decreased macrophage clustering and fibrosis. PAO inversely correlated with T, with levels <3.76 nM predicting abnormal PAO with >80% sensitivity and specificity. MetS causes progressive lung injury via macrophage dysregulation, inflammation, and peribronchiolar fibrosis. T deficiency is central, as hormone administration improved lung function and histology. Immune-driven mechanisms, including Th2/Th17 cytokines and epithelial-mesenchymal transition markers, likely contribute. T's anti-inflammatory and antifibrotic effects may involve cAMP signaling. Clinically, assessing T and metabolic status is crucial, and T therapy may help mitigate lung consequences of MetS.

Metabolic disorders, including obesity, metabolic syndrome (MetS), and diabetes, may have a relevant impact on pulmonary health. These conditions could not only impair baseline respiratory function but also foster systemic inflammatory and hormonal imbalances that heighten vulnerability to both chronic lung diseases and acute respiratory insults (1). The COVID-19 pandemic has further documented these interconnections, placing patients with metabolic dysfunction at elevated risk for pulmonary complications. Diabetes constitutes a common comorbidity among patients with severe SARS-CoV-2 (2) and induces a marked worsening of pulmonary disease and higher mortality (3-5).

Cross-sectional and longitudinal data showed that lung function was impaired in diabetes and prediabetes. An additional “dose effect” was demonstrated, wherein participants with diabetes exhibited worse lung function when their glycosylated hemoglobin was 7% or more (6). A link between MetS and lung function impairment has also been documented (7, 8). Recently, a study showed that patients with MetS experience reductions in both forced expiratory volume and forced vital capacity, nearly doubling the prevalence of lung dysfunction compared to individuals without the syndrome (9). Obesity and MetS are strongly associated with decreased lung volumes and impaired respiratory mechanics. Components such as abdominal obesity, hypertension, and dyslipidemia each contribute to diminished pulmonary performance, largely driven by persistent low-grade inflammation (10).

COVID-19 disproportionately impacted individuals with obesity, with research showing a markedly higher risk of severe pneumonia in those with a body mass index >30, and a greater likelihood of requiring invasive ventilation with a body mass index >35 (11). In a meta-analysis, diabetes mellitus was the best predictors of SARS-CoV-2-associated mortality rate in an age- and sex-dependent manner, followed by chronic pulmonary obstructive diseases and malignancies (12).

Testosterone (T) could be considered not only a regulator of male sexual function, but also of metabolic and pulmonary functions (13). Hypogonadism, commonly found in men with obesity, diabetes, or chronic respiratory disease (4, 14), has been associated with decreased respiratory muscle strength and poorer lung performance (15). A retrospective study conducted in Italy included 31 male patients admitted to a respiratory intensive care unit with confirmed SARS-CoV-2 pneumonia (16). The results originally demonstrated that patients who experienced worse respiratory outcomes had significantly lower total T and calculated free T levels at admission compared to those with more favorable clinical courses. Lower T levels were also strongly correlated with elevated inflammatory markers. Statistical analysis showed that for each 1 nmol/L decrease in total T the odds of a worse outcome raised by approximately 42% (16). These data have been later confirmed by other authors (17, 18). In detail, the decreased T levels are often independently linked to a longer hospital stay and a greater need for respiratory support, regardless of age or comorbidity status (17, 18).

MetS is a cluster of metabolic and cardiovascular (CV) risk factors (including glucose intolerance, visceral obesity, dyslipidemia, and hypertension) that increase the odds for developing diabetes and major adverse CV events. In men, MetS is often associated with T deficiency and secondary hypogonadism (19). Clinical evidence has shown a link between MetS and lung disease and demonstrated that MetS factors can predict lung function deterioration (1). A chronic, systemic, and tissue-specific meta-inflammation triggered by MetS has been demonstrated in adipose tissue (20), liver (21), skeletal muscle (22, 23), hypothalamus (24), and pancreas (25). It is therefore conceivable that a similar picture could be present also in the lung. However, the condition complexity and the lack of valid experimental models have limited research in the field.

Our group developed a non-genomic preclinical animal model of MetS, obtained by feeding New Zealand White rabbits with a high-fat diet (HFD) for 12 weeks (21, 26-29). This model faithfully also reproduces the phenotype of human MetS, including hypertension, hyperglycemia, dyslipidemia, glucose intolerance, and visceral fat accumulation. New Zealand White rabbits are recognized as the experimental model primary choice, since they consistently show exhaustive HFD-induced MetS hallmarks after 12 weeks of HFD (30). Interestingly, this model also displays hypogonadotropic hypogonadism and altered levels of inflammatory cytokines and organ-specific inflammation (21).

In the HFD-induced rabbit model of MetS, visceral adipose tissue (VAT) develops insulin-resistant preadipocytes with impaired lipid handling, mitochondrial dysfunction, and defective adipogenesis, along with hypothalamic inflammation (24, 31). Adipocyte insulin resistance is a central driver of MetS and is associated with elevated triglycerides and cholesterol (32-34). VAT accumulation, rather than overall body weight, underlies hyperglycemia and hyperlipidemia, whereas increased triglycerides promote ectopic lipid deposition and inflammation in the liver, further exacerbating insulin resistance (35, 36).

Using this animal model, an anti-inflammatory effect of T treatment has been demonstrated in several tissues, including liver (21), bladder (27), prostate (37), and hypothalamus (38). Hence, it will be intriguing to test if even in lung T might exert anti-inflammatory and antifibrotic effects.

The endpoint of this study was to better understand the pathogenesis of lung dysfunction in MetS, analyzing the inflammatory and tissue remodeling process in the lung of the aforementioned MetS model. Given that the most severe pulmonary dysfunctions during the COVID-19 pandemic were observed in patients with metabolic comorbidities, and that hypogonadism was identified as a hallmark of poor clinical pulmonary outcomes, we conducted a series of preliminary studies.

We aimed at initially providing an insight into the preventive role of in vivo T administration in HFD-induced metabolic and pulmonary abnormalities. These investigations used morphological and molecular analyses to evaluate the effects of experimental metabolic syndrome and hypogonadism, as well as the impact of its pharmacological treatment. The preliminary results were highly encouraging. Consequently, in a small subset of the cohorts, we conducted functional studies by measuring the airway opening pressure. The results were compelling and consistent with the aforementioned observations, suggesting a beneficial effect of chronic T therapy. A primary limitation of this experimental series was that T administration was initiated before the onset of the disease. Consequently, this experimental design does not fully reflect the clinical reality in humans, where treatment is typically commenced after the disease state has already been established.

Therefore, we designed a second set of experiments to verify the following endpoints: (1) after 6 weeks of HFD, the primary hallmarks of MetS and a hypogonadal state begin to emerge; and (2) T administration at this specific time point is capable of reversing pulmonary alterations, including functional impairments, once the disorder has become manifest. To establish the 6-week time point as the baseline disease stage for initiating acute T treatment, biochemical and metabolic data from controls and HFD groups at week 6 were included. Functional assessment, immunohistochemical analyses, and molecular evaluation were then performed to determine the effects of the 6-week therapeutic T treatment, in comparison with both early-stage disease (6-week HFD) and advanced metabolic syndrome with lung dysfunction (12-week HFD).

## Materials and methods

All experimental procedures were conducted using the facilities of the Department of Biomedical Experimental and Clinical Sciences “Mario Serio” and Department of Experimental and Clinical Medicine, and those of CE.S.A.L. (Centro Stabulazione degli Animali da Laboratorio), and NEUROFARBA Department, University of Florence, Italy.

### Experimental plan

Eighteen-week-old young adult male New Zealand White rabbits (Charles River, Calco, Lecco, Italy), weighing approximately 3 kg, were individually caged under standard conditions in a temperature and humidity-controlled room on a 12-hour light/dark cycle. Food and water were provided ad libitum throughout the study.

As a preliminary investigation to evaluate the effects of T in a 12-week high-fat diet (HFD) model, a chronic 12-week in vivo treatment protocol was implemented. This initial phase was designed to assess the preventive effects of T and to validate the model for subsequent evaluation under an acute therapeutic regimen. Following stabilization on a regular diet (RD), 31 animals were randomly assigned to 3 experimental groups:

- RD12W—Control rabbits maintained on an RD for 12 weeks (n = 12);
- HFD12W—Rabbits fed an HFD (RD supplemented with cholesterol and peanut oil) for 12 weeks (n = 12);
- HFD + T12W—Rabbits fed a HFD and treated from the beginning of dieting with injectable T administration (30 mg/kg/week, IM) for 12 weeks (n = 7).

MetS evaluation, lung androgen receptor expression, and lung molecular analyses were carried out for these groups to test the general hypothesis that a preventive 12-week T administration would be beneficial. The initial findings were promising; accordingly, we assessed pressure of airway opening in a small subset of the cohorts (n = 3 per group).

The second phase of the study aimed to evaluate the therapeutic effects of testosterone administered over the final 6 weeks of a 12-week HFD protocol. After stabilization on a regular diet, an additional 13 rabbits were randomly assigned to the following groups:

- HFD6W—Rabbits fed an HFD and euthanized after 6 weeks (n = 6);
- HFD12W + T6W—Rabbits fed an HFD for 12 weeks and treated with injectable T administration (30 mg/kg/week, IM) during the last 6 weeks only (n = 7).

MetS evaluation, serum free long-chain fatty acids quantification by GC-MS analysis, pressure of airway opening functional analysis, RAM11 and picrosirius red histological analyses, and pulmonary molecular analyses were carried out for the second phase groups. These data were also obtained from RD12W and HFD12W groups to assess progression of the disease and the ability of 6-week T administration in ameliorating the outcomes.

In accordance with the 3Rs principles of animal research (Replacement, Reduction, and Refinement), efforts were made to minimize animal use and avoid unnecessary sacrifice. Rather than including an additional control group to be euthanized at 6 weeks, the existing RD12W group was evaluated for biochemical and metabolic parameters at the 6-week time point, where available (RD6W; N = 6), for MeTs assessment. This approach reduced the total number of animals required. Because rabbits maintained on a regular diet did not develop metabolic or lung dysfunction after 12 weeks, HFD6W data were then compared against RD12W values.

Testosterone enanthate (Testoviron; A.I.C. no. 002922060; Bayer S.p.A., Milan, Italy) was used in a lipophilic vehicle consisting of castor oil, with benzyl benzoate (0.34 g/L) serving as a cosolvent. The vehicle of this preparation has historically been used for T administration in previous experiments in our laboratory, without eliciting any observable effects and consistently showing no detectable biological activity. Therefore, it was not included in the current experimental design. The diet specifications are as follows:

**Table:** 

Composition	Regular diet (RD)	High-fat diet (HFD)
Water (%)	12.0	12.0
Protein (%)	16.5	12.6
Vegetable-derived fat (%)	3.5	6.0
Animal-derived fat (%)	0.0	0.5
Fiber (%)	15.5	21.2
Ash (%)	8.5	9.2
NFE (%)	44.0	38.5

NFE (nitrogen-free extract) represents the readily digestible carbohydrate fraction and includes sugars, starch, and soluble carbohydrates.

Rabbits were sacrificed by a lethal dose of sodium thiopental (200 mg/kg IV), and prostate, seminal vesicles, and lung specimens were harvested and appropriately stored at −80 °C for the subsequent analyses.

### Ethical Committee and Ministry of Health approval

Animal handling and protocols complied with Animal Welfare Body of the University of Florence, Florence, Italy, in accordance with the Italian Ministerial Law n. 26/2014. The study complied with the Ministry of Health authorization n. 724/2022-PR. Animal experiments conformed to the Animal Research: Reporting of In Vivo Experiments guidelines (RRID:SCR_018719). Animals were included after stabilization on a regular diet; animals with illness or abnormal baseline parameters were excluded. In the preventive phase (phase 1), rabbits were randomly assigned to RD, HFD, or HFD plus T. In the therapeutic phase (phase 2), additional rabbits were randomly assigned to HFD or HFD with T during the final 6 weeks. Outcome measures included biochemical and metabolic parameters, lung function, histology, and molecular analyses. Group allocation, conduct of the experiments, outcome assessments, and data analysis were performed blindly wherever applicable. The study adhered to the 3Rs principles to minimize animal use and unnecessary sacrifice.

### MetS evaluation

Blood samples were obtained from the marginal ear vein at week 6 or 12, depending on the time of culling. All blood samples were collected in standard conditions, before 10 Am after an overnight fasting. The blood was immediately centrifuged at 2000*g* for 20 minutes at 4 °C and collected plasma stored at −80 °C until assayed.

An oral glucose tolerance test (OGTT) was performed in accordance with the published method (26). Briefly, after an overnight fast, a 50% glucose solution was orally administered to the animals at a dose of 1.5 g/kg. Blood samples were collected via the marginal ear vein before and 15, 30, and 120 minutes after glucose loading. The incremental area under the curve was calculated by using the GraphPad Prism software (v. 8.4; Insight Partners, New York, NY).

Mean arterial blood pressure (MAP) was measured by using a polyethylene catheter inserted into a femoral artery at week 12, after ketamine (10 mg/kg, IV) and sodium thiopental (50 mg/kg, IV) sedation. Plasma glucose, total cholesterol, and triglycerides were measured using an automated system (Cobas 8000; Roche Holding AG, Basel, Switzerland). The analytical performance characteristics of the assays were as detailed here:

- Glucose: lower limit of detection (LOD) 0.02 g/L; intra-assay coefficient of variation range 0.5-1.3%
- Total cholesterol: LOD 3.86 mg/dL; coefficient of variation range 0.6-0.8%
- Triglycerides: LOD 8.85 mg/dL; coefficient of variation range 0.7-0.9%

Plasma T levels were measured by ECLIA (ElectroChemiLuminescence ImmunoAssay) using a commercially available immunoassay validated for human samples (Elecsys Testosterone II Kit; Roche Holding AG; Cat# 05200067; RRID:AB_2783736) with an automated chemiluminescence system (Cobas Integra 8000; Roche Holding AG). Testosterone LOD and coefficient of variation range were 0.087 nmol/L and 1.3% to 5.7%, respectively. Because steroid hormones are structurally conserved across mammalian species, including rabbits, cross-species detection by antibody-based assays is expected. To confirm suitability for rabbit plasma, assay performance was verified through serial dilution analysis, which showed linearity and parallelism with the calibration curve, indicating absence of significant matrix interference.

### Molecular analyses

TRIzol reagent (Life Technologies, Paisley, UK) and RNeasy Mini Kit (Qiagen, Hilden, Germany) were used to isolate total RNA from rabbit liver specimens. cDNA synthesis was carried out using the iScript cDNA Synthesis Kit (Bio-Rad Laboratories, Hercules, CA). Semiquantitative real-time RT-PCR amplification and detection was performed with SsoAdvanced Universal SYBR Supermix and a CFX96 Duet Real-Time PCR Detection System (both Bio-Rad Laboratories). Specific PCR primers for rabbit target genes were designed on sequences available at the National Center for Biotechnology Information GenBank (RRID:SCR_002760) or Ensembl Genome (RRID:SCR_002344) and are reported in Table 1. The *18S* ribosomal RNA subunit was used as the housekeeping gene for the relative quantitation of the target genes based on the comparative threshold cycle 2^−ΔΔCt^ method (39), with some modifications. In detail, we used the control groups as the calibrator in each analysis, so that the calculations would provide the fold-change of the other groups relative to controls.

**Table 1:** List of primer sequences

Gene	Forward sequence (5′-> 3′)	Reverse sequence (5′-> 3′)
*18S*	GGCCGTTCTTAGTTGGTGGA	AGCATGCCAGAGTCTCGTTC
*AR*	CCGTAACTTGCATGTGGATG	GCTGTACATCCGGGACTTGT
*CD11c*	CTTTGACCTTGCCCTTGACC	ACCAGGGAGAAGTTGAGACG
*CD206*	ATTATGTGTCCTGGGCCACA	TGATGCTGCTGTTATGTCGC
*COL1A1*	ACTGGATTGACCCCAACCA	TTGCCCCAGTGTCCATGTC
*COL3A1*	CATTGGCCCTGTTTGCTTTT	GTTGGTCACTTGTACTGGTTGACA
*COX2*	AGTGTGCGATGTGCTCAAAC	AAAAGCAGCTCTGGGTCAAA
*CREB1*	ACAGTATGCACAGACCACGG	GCTGTGCGGATCTGGTATGT
*CREM*	GAGAGAGAAGGAACACCGCC	GCCTGTAGTCCCTGAACACC
*EPAC1*	AGGGACCTCTTCTACAGCGT	CCAGTACTGCAGCTCGTTGA
*ET1*	AGCGAGTAGCAGCTCCAAAG	CCTGAGCCTGTCAGTGCATA
*ETRA*	AGGGGTGAACAGCACAAAAC	ATGTTCACTGAGGGCAATCC
*ETRB*	CGTCTGCACCTGCTGAAATA	AACACGAGGCAGGATACCAC
*FN1*	CCTGCACCAAGAATTGGTTT	TACGATCGGAGCGTCTCTTT
*FOXP3*	CTTTCACCTACGCCACCCTC	GTAGCAGGGTGGTTTCGGAA
*GATA3*	AGGCAGGGAGTGTGTGAACT	CGTCGTGGTCTGACAGTTTG
*IL1β*	CCACAGTGGCAATGAAAATG	AGAAAGTTCTCAGGCCGTCA
*IL6*	TGAAGAAGCCACCCTCAAGC	AGAGCCCATGAAATTCCGCA
*IL10*	AGAACCACAGTCCAGCCATC	TTTTCACAGGGGAGAAATCG
*IL12p35*	TCGGAAAAGTCAGAGGCGAC	CTCTGCATCAACTTCCCGGT
*LOX1*	GATGCCCCACTTGTTCAGAT	CAGAGTTCGCACCTACGTCA
*eNOS*	GCACAGTGATGGCAAAGAGA	TCGAGGGACACCACATCATA
*PDE5*	GCATCTGAATTTGACCGGCC	GTGCCCTCAGAGTCCTTGAC
*PKA*	TTTGCAGACCAACCAATTCA	GTTGTGGCAAACCATTTGTG
*PKG1*	TGGATGACGTTTCCAACAAA	CACTATGTGGCGCTTCTTGA
*RORγt*	TACACGGCGCTTGTTCTCAT	TGCAGCTGTTCCACTTTCCT
*αSMA*	ACTGGGACGACATGGAAAAG	TACATGGCTGGGACATTGAA
*SNAI1*	TGGGTTTGCGTATCCAGAGG	GTCTGTCGCTTTTGTCCTGC
*SNAI2*	TACAGCCCCATAACCGTGTG	ATGAGGTGTCGGATGGAGGA
*TBET*	GATGTTCCCGTTCCTGTCGT	TTTCCACACTGCACCCACTT
*TGFβ1*	CTTCCGCAAGGACCTGGG	CGGGTTGTGCTGGTTGTAC
*TGFβ3*	TCCAACTTGGGTCTGGAGAT	AGGATGAGGTGAGGGTTGTG
*TLR2*	CCGCGGGTTCCCCAGGTTG	GGATCTGGAGCGCCCATCGC
*TLR4*	GCGGGTGGAGCTGTATCGCC	CTTGGGTTCAGCCGGGCAGG
*TNFα*	CGTCTCCTACCCGAACAAGG	GGTACTCAGGCTGGTTGACC

### Serum free long-chain fatty acids quantification by GC-MS analysis

Free long-chain fatty acids (LCFAs) were analyzed using a previously described GC-MS protocol (40). Briefly, just before the analysis, each sample was thawed and the free fatty acids were extracted as follows: an aliquot of 200 μL of serum sample was added to 10 μL of internal standard mixture, 100 μL of tert–butyl methyl ether, and 20 μL of 6 M HCl, 0.5 M NaCl solution in a 0.5-mL centrifuge tube. Afterwards, each tube was stirred in a vortex for 2 minutes and centrifuged at 6000*g* for 5 minutes, and the solvent layer was transferred to a vial with a microvolume insert and analyzed.

### Functional analyses

Before culling, rabbits underwent measurement of airway resistance to ventilation (pressure of airway opening [PAO]), a functional parameter related to fibrosis-induced reduced lung compliance, using a constant-volume mechanical ventilation method (41). Briefly, after anesthesia, rabbits were surgically inserted with a steel cannula into the trachea and then ventilated with an animal respirator (Ugo Basile, Bologna, Italy), set to deliver a volume of 40 mL at a rate of 55 strokes/min.

Changes in pulmonary resistance to ventilation were recorded for at least 3 minutes by a highly sensitive pressure transducer (P75 type 379; Hugo Saks Elektronik, March-Hugstetten, Germany) connected to a polygraph (Harvard Apparatus, Holliston, MA), with the following settings: gain, 2-0; chart speed, 25 mm/s. In each rabbit, PAO measurements (expressed in millimeters on the graph) were performed on at least 100 consecutive breath traces after respiratory stabilization, and the observed values were then averaged.

### Histochemical analyses

After fixation in 10% neutral buffered formalin (Sigma-Aldrich, St. Louis, MO), lung samples were embedded in paraffin and sectioned into 5-μm slices with a standard microtome. Immunohistochemical analyses were performed to evaluate the expression of androgen receptor (AR) and macrophage marker (RAM11). For AR detection, tissue sections were deparaffinized, rehydrated, and incubated with a primary rabbit polyclonal anti-AR antibody (1:50; Santa Cruz Biotechnology, Dallas, TX; Cat# sc-816; RRID:AB_1563391). RAM11 expression was assessed using a mouse monoclonal anti-RAM11 antibody (1:80; Agilent, Santa Clara, CA; Cat# M0633; RRID:AB_3227442).

Sections were incubated overnight at 4 °C with the primary antibody, followed by incubation with a biotinylated goat anti-mouse secondary antibody (Millipore, Burlington, MA; Cat# 21538, RRID:AB_916360), and subsequently with a streptavidin-peroxidase complex (Millipore). The immunoreactivity was visualized using 3,3′-diaminobenzidine tetrahydrochloride (Merck KGaA, Darmstadt, Germany) as the chromogen. Negative controls were included by omitting the primary antibody to confirm signal specificity. For quantitative analysis, the number of RAM11-positive cellular clusters containing 3 or more cells, as well as the number of individual alveolar macrophages, were counted in 10 fields per slide and expressed as the mean ± SEM.

Fibrosis assessment was performed through histological staining using the picrosirius red stain kit (Bio-Optica, Milan, Italy), following the manufacturer's protocol. In brief, deparaffinized and rehydrated lung sections were stained with the picrosirius red solution for 50 minutes, rinsed with appropriate reagents and distilled water, then dehydrated through graded ethanol and cleared in xylene before mounting, as previously described (42). Fibrotic areas were quantified using ImageJ software (RRID:SCR_003070) by calculating the percentage of picrosirius red-stained area within randomly selected bronchioles or parenchymal regions at 200× magnification. Quantitative evaluations were performed on at least 3 nonoverlapping fields per section, randomly selected within the lung parenchyma or bronchiolar regions, depending on the specific analysis. Immunohistochemical and histological analyses were performed on 3 paraffin sections per animal, obtained from serial sections collected at standardized depths of the tissue block, with a spacing of 50 µm between consecutive sections. All slides were evaluated in a blinded manner and collected using a Nikon Microphot-FXA microscope (Nikon, Tokyo, Japan). To avoid overlapping areas, regions of interest were identified at low magnification and nonadjacent fields were selected at fixed magnification. The mean value obtained from the analyzed fields of each animal was used for statistical analysis.

### Three-dimensional analysis of optically cleared lung biopsies with light-sheet microscopy

Formalin-fixed, paraffin-embedded lung tissues were processed adapting the iDISCO + framework (43) and, unless specified otherwise, all steps were performed at room temperature, with shaking, for 40 minutes. First, after removing manually as much paraffin as possible from the samples with a blade, tissues were deparaffinized by incubation with other paraffin at 60 °C until all the paraffin had melted. Subsequentially, to ensure complete paraffin removal, the samples were washed several times in fresh xylene until the solution remained clear. After an overnight incubation in fresh xylene and three washes with 100% methanol (MeOH) samples were bleached overnight at 4 °C in freshly prepared 5% hydrogen peroxide (H_2_O_2_) in MeOH, without shaking. For RAM11 immunolabeling, tissues were first rehydrated through a descending MeOH series in ddH_2_O (60%, 40%, 20%), washed in PBS, and then twice with 0.2% Triton X-100 in PBS (PBST). A permeabilization step was then performed by incubating the tissues at 37 °C for 24 hours in a permeabilization solution containing 0.2% (v/v) TritonX-100, 20% (v/v) dimethyl sulfoxide (DMSO), 2.3% (w/v) glycine (Sigma-Aldrich) in PBS. Blocking was carried out for 24 hours at 37 °C in 0.2% (v/v) Triton X-100, 0.01% (w/v) sodium azide (Sigma-Aldrich), 0.2% (w/v) gelatin porcine skin (G1890; Sigma-Aldrich) in PBS.

The samples were then incubated for 5 days at 37 °C with a mouse monoclonal anti-RAM11 primary antibody (Agilent), diluted 1:200 in a buffer composed of 0.2% (v/v) Tween-20, 0.001% w/v Heparin (H5515-100KU; Sigma-Aldrich) in PBS (PBSTw-H). After 24-hour washes in PBSTw-H, the samples were incubated for 5 days at 37 °C in a 1:1000 dilution of a goat anti-mouse Alexa Fluor 647 secondary antibody (ab150115; Abcam, Cambridge, UK) prepared in PBSTw-H.

Following another 24 hours of washes in PBSTw-H, samples were dehydrated through an increasing MeOH series in ddH_2_O (20%, 40%, 60%, 80%, 100% MeOH) and stored overnight in fresh 100% MeOH. For refractive index (RI) matching, the samples were incubated for 3 hours in a solution of 66% (v/v) dichloromethane (270997-250 mL; Sigma-Aldrich) and 33% (v/v) MeOH, followed by two 30-minute washes in 100% dichloromethane.

Finally, optical clearing and storage were achieved by transferring the samples in dibenzyl ether (108014; Sigma-Aldrich). Cleared samples were imaged with a custom-made high-resolution light-sheet microscope described in detail elsewhere (44). Raw images were stitched together using ZetaStitcher (RRID:SCR_026193).

Given the high signal-to-background ratio of the raw data, RAM11-positive cells were effectively detected by direct thresholding. We identified as cell clusters all segmented volumes larger than 30,000 cubic microns. Cell segmentation and cluster identification was performed with custom scripts in Python. Orthogonal views and volumetric renderings were generated using FIJI-ImageJ (RRID:SCR_002285) to visualize RAM11-positive cells and clusters.

### Statistical analyses

Results are reported as mean ± SEM. Statistical analysis was performed using Shapiro-Wilk test of normality and, to evaluate differences between groups (with *P* < .05 considered as significant), as follows: (1) normally distributed parameters were tested for variance with Levene test (F-test if only 2 groups) and then Welch *t*-test (for 2 groups) and either parametric 1-way ANOVA or Welch ANOVA test (for 3 or more groups), followed by post hoc Tukey honestly significant difference test or Games-Howell test, respectively; (2) not normally distributed parameters were tested with 1-way nonparametric Kruskal-Wallis ANOVA followed by post hoc Mann-Whitney analysis. Correlations were evaluated by Spearman bivariate correlation analyses and by multivariate analyses. Statistical analysis was performed with Statistical Package for the Social Sciences (v.31.0.1; IBM SPSS, Armonk, NY) or GraphPad Prism (v.8.4.3; GraphPad Software).

## Results

### PHASE 1

This section of results represent the preliminary study to test the general hypothesis that a preventive 12-week T administration would benefit from a metabolic and pulmonary molecular perspective, compared to HFD12W and controls (groups included: RD12W, HFD12W, HFD + T12W).

#### Phase 1—biochemical and metabolic analyses in HFD for 12 weeks with or without 12-week testosterone administration

Table 2 reports the indicated parameters in the RD12W and before (HFD12W) and after (HFD + T12W) 12 weeks of T administration. As expected (21, 26-29), HFD induced all the typical MetS alterations, although HFD animals exhibited a reduction in total body weight compared to controls, which may reflect HFD-induced skeletal muscle hypotrophy, as previously suggested (45, 46). In line with this, our earlier studies reported that HFD rabbits displayed reduced physical endurance relative to RD animals (29), accompanied by skeletal muscle alterations induced by the HFD (23).

**Table 2:** Biochemical and metabolic parameters in the experimental rabbits from phase 1 groups

Analyses	RD (n = 12)	HFD12W (n = 12)	HFD + T12W (n = 7)
Body weight (g)	3953.92 ± 36.77	3438.83 ± 67.20***	3734.14 ± 132.96
Glycemia (g/L)	0.92 ± 0.05	2.23 ± 0.27**	1.31 ± 0.15^
OGTT (iAUC)	160.11 ± 5.59	204.75 ± 10.82**	176.23 ± 22.41
Cholesterol (mg/dL)	31.50 ± 3.32	2273.25 ± 316.69***	759.86 ± 93.06*** ^^
Triglycerides (mg/dL)	59.33 ± 5.08	387.58 ± 125.38***	90.00 ± 19.66^^
MAP (mm Hg)	81.94 ± 3.77	141.75 ± 8.52***	116.04 ± 5.19** ^
VAT Weight (% of BW)	0.964 ± 0.024	1.049 ± 0.033*	0.208 ± 0.069*** ^^^
Testosterone (nmol/L)	10.40 ± 2.06	2.99 ± 0.51***	34.76 ± 7.28* ^^^
Prostate weight (% of BW)	0.019 ± 0.001	0.013 ± 0.002**	0.024 ± 0.002^^^
S.V. weight (% of BW)	0.019 ± 0.002	0.011 ± 0.001**	0.061 ± 0.006*** ^^^

Abbreviations: BW, body weight; iAUC, incremental area under the curve of glucose blood level during oral glucose tolerance test (OGTT); MAP, mean arterial pressure; S.V., seminal vesicles; VAT, visceral adipose tissue.

Results are reported as mean ± SEM. To evaluate differences between groups, with *P* < .05 considered as significant, all biomarkers were analyzed by either parametric Welch ANOVA test followed by post hoc Games-Howell test (for normally distributed parameters) or 1-way nonparametric Kruskal-Wallis ANOVA followed by post hoc Mann-Whitney analysis (for not normally distributed parameters).

^*^ *P* < .05, **P < .01, ***P < .001 vs RD12W; ^*P* < .05, ^^*P* < .01, ^^^*P* < .001 vs HFD12W.

Testosterone treatment significantly improved several of the observed biochemical and metabolic parameters, namely glucose, cholesterol, triglycerides level, MAP, and VAT weight. The 12-week in vivo T administration also remarkably reduced OGTT.

Moreover, T administration counteracted the HFD-induced hypogonadism and the decreased weight of androgen-dependent tissues, such as prostate and seminal vesicles (Table 2).

Functional studies conducted in a limited number of animals (n = 3) from each group showed that chronic T exposure normalized the pressure of airway opening (RD12W = 17.53 ± 0.71 mm, *P* < .05 vs HFD12W; HFD12W = 29.76 ± 1.86 mm; HFD + T12W = 18.73 ± 1.64 mm, *P* < .05 vs HFD12W).

#### Phase 1—immunohistochemical and molecular androgen receptor expression in lung tissue

**Figure 1:**
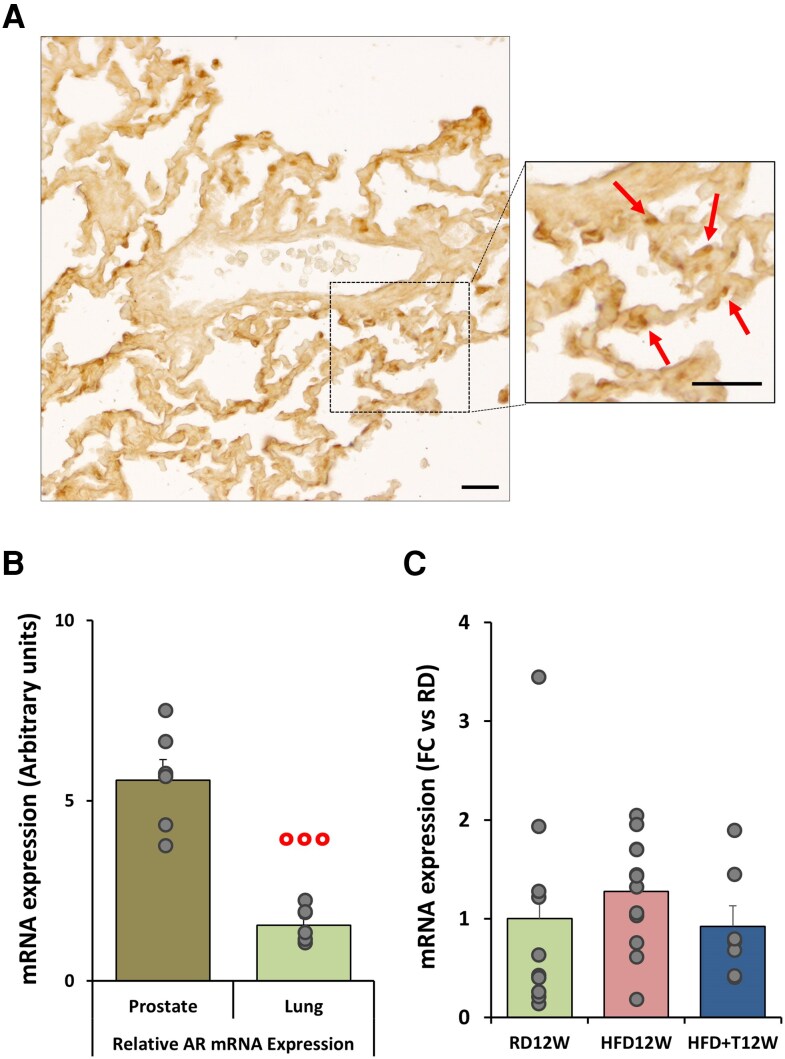
Immunohistochemical and molecular androgen receptor expression. (A) Representative image of an RD12W lung section. The inset shows a higher magnification of the boxed area, where arrows indicate AR positivity, localized in the nuclei of epithelial cells. Scale bar = 20 µM. (B) Relative mRNA expression of AR in prostate and lung homogenates (n = 6 each). Results are reported as mean ± SEM. (C) Semiquantitative lung mRNA expression of AR in the RD12W (n = 10), HFD12W (n = 12), and HFD + T12W (n = 7) experimental groups. Results are expressed as mean ± SEM and reported as fold-change vs RD12W. To evaluate differences between groups, with *P* < .05 considered as significant, data were analyzed by either Welch *t*-test (B) or 1-way nonparametric Kruskal-Wallis ANOVA (C; *P* = .253). °°° *P* < .001 vs prostate.

In Fig. 1A, we illustrate the AR immunolocalization in a representative rabbit lung section. AR is expressed at pulmonary levels, with a specific nuclear localization in epithelial cells, mostly likely type 2 pneumocytes, as previously reported (47). *AR* mRNA expression was also evaluated in lung homogenates and relatively compared to the mRNA expression in the prostate, a well-known androgenic-dependent tissue (Fig. 1B). We confirm that lung tissues express *AR*, although at an extent >3-fold lower than prostate. Last, we evaluated the lung *AR* mRNA expression in RD12W, HFD12W, and HFD + T12W animals, without observing statistically significant differences among these experimental groups (Fig. 1C).

#### Phase 1—molecular analyses of pro-fibrotic and pro-inflammatory markers of lung tissue in HFD for 12 weeks with or without 12 weeks of T administration

**Figure 2:**
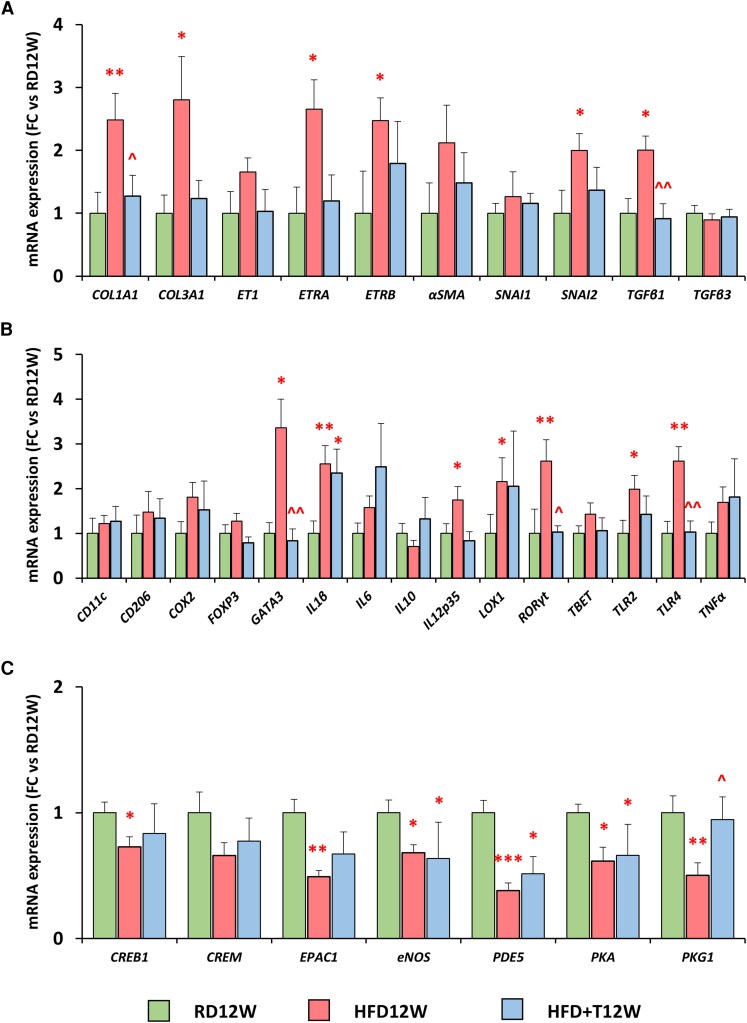
Molecular analyses of lung homogenates. (A-C) Semiquantitative lung mRNA expression of pro-fibrotic, pro-inflammatory, and NO/cGMP and cAMP signaling, respectively, in the RD12W (n = 10), HFD12W (n = 12), and HFD + T12W (n = 7) experimental groups. Results are expressed as mean ± SEM and reported as fold-change vs RD12W. To evaluate differences between groups, with *P* < .05 considered as significant, all biomarkers were analyzed by either one-way non-parametric Kruskal-Wallis ANOVA followed by post hoc Mann-Whitney analysis (for not normally distributed parameters) or 1-way parametric ANOVA test followed by post hoc Tukey honestly significant difference (HSD) test (for normally distributed parameters). *P < .05, **P < .01, ***P < .001 vs RD12W; ^*P* < .05, ^^*P* < .01 vs HFD12W.

Figure 2 shows the mRNA expression of several lung target genes in the aforementioned experimental groups. HFD induced statistically significant increase in several (*COL1A1*, *COL3A1*, *ETRA*, *ETRB*, *SNAI2*, and *TGFβ1*) but not all (*ET1*, *αSMA*, *SNAI1*, and *TGFβ3*) pro-fibrotic markers (Fig. 2A). Likewise, the mRNA expression of several pro-inflammatory genes was also upregulated by HFD (eg, *GATA3*, *IL1β*, *IL12p35*, *LOX1*, *RORγt*, *TLR2*, *TLR4*) (Fig. 2B). The 12-week T treatment normalized most of these genes mRNA expression, showing a consistent decrease of the expression of pro-fibrotic and inflammatory markers (Fig. 2A-B). Finally, we evaluated the mRNA expression of cGMP and cAMP signaling pathways, finding that HFD induced a significant downregulation of various genes of both pathways. T treatment caused a partial recovery for PKA downstream effectors (*CREB1*, *CREM*, *EPAC1*) and a full recovery for *PKG1* mRNA expression (Fig. 2C).

### PHASE 2

This section of results represent the second phase of the project, to assess progression of the disease and the ability of 6-week T administration in ameliorating biochemical, functional, immunohistochemical, and molecular outcomes. In particular, (1) the progressive changes during MetS development and following acute 6-week testosterone treatment (groups included: RD at 6 and 12 weeks, HFD at 6 and 12 weeks, and HFD12W + T6W); and (2) the efficacy of therapeutic 6-week T administration after early- and late-stage MetS and lung dysfunction (groups included: RD12W, HFD6W, HFD12W, and HFD12W + T6W).

#### Phase 2—biochemical and metabolic analyses in HFD for 6 or 12 weeks with or without 6 weeks of T administration

**Figure 3:**
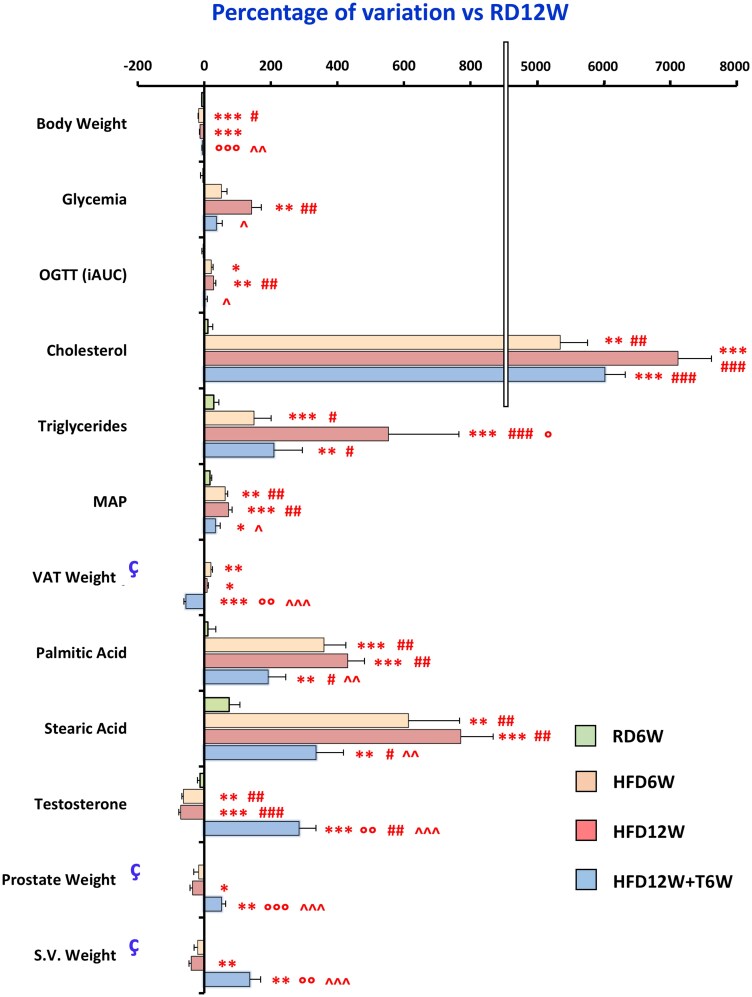
Effects of preventive in vivo testosterone treatment on biochemical and metabolic parameters in the RD6W (n = 6), HFD6W (n = 6), HFD12W (n = 12), and HFD12W + T6W (n = 7) experimental groups, expressed as percentage of variation vs RD12W (n = 12). Results are reported as mean ± SEM; data not available for RD6W. Data from RD12W and HFD12W, previously presented in Table 2, are hereby reported for comparisons. To evaluate differences between groups, with *P* < .05 considered as significant, all biomarkers were analyzed by: (A) for normally distributed parameters either 1-way parametric ANOVA test followed by post hoc Tukey honestly significant difference (HSD) test or parametric Welch ANOVA test followed by post hoc Games-Howell test, according to Levene variance test; (B) for not normally distributed parameters 1-way nonparametric Kruskal-Wallis ANOVA followed by post hoc Mann-Whitney analysis. *P < .05, **P < .01, ***P < .001 vs RD12W; #*P* < .05, ##*P* < .01, ###*P* < .001 vs RD6W; °*P* < .05, °°*P* < .01, °°°*P* < .001 vs HFD6W; ^*P* < .05, ^^*P* < .01, ^^^*P* < .001 vs HFD12W.

We next assessed the ability of T to exert a therapeutic effect after the induction of the MetS condition. To this aim, we first investigated the effects of a 6-week T administration following 6 weeks of HFD (HFD12W + T6W), also comparing the obtained data with a group of animals sacrificed after 6 weeks of HFD (HFD6W) to establish the advancement of the disease at this earlier stage. The RD12W group was also evaluated for available biochemical and metabolic parameters collected at the 6-week time point (RD6W), for MetS assessment. No significant differences were observed between RD6W and RD12W (Fig. 3).

After 6 weeks, HFD was already able to induce most of the MetS abnormalities observed at 12 weeks, compared to RD at 6 and 12 weeks, namely dyslipidemia, increased MAP, VAT weight, and incremental area under the curve of OGTT, thus indicating that after 6 weeks the MetS condition was already established (Fig. 3). Interestingly, LCFA plasma concentrations, such as palmitic and stearic acids, were significantly increased in a time-dependent manner in HFD animals.

Even at this earlier stage, HFD was also able to induce an overt condition of hypogonadism with a significant reduction of T level associated with a reduction of prostate and seminal vesicles weight (Fig. 3). Notably, the 6-week T treatment (starting from week 6 to 12) was able to counteract not only the hypogonadal state, but also most of the negative effects induced by the HFD, having significant improvement on glycemia, OGTT, MAP, VAT weight, and LCFA parameters, compared to HFD12W.

#### Phase 2—functional analyses of lung resistance to inflation in HFD with or without 6 weeks of T administration

**Figure 4:**
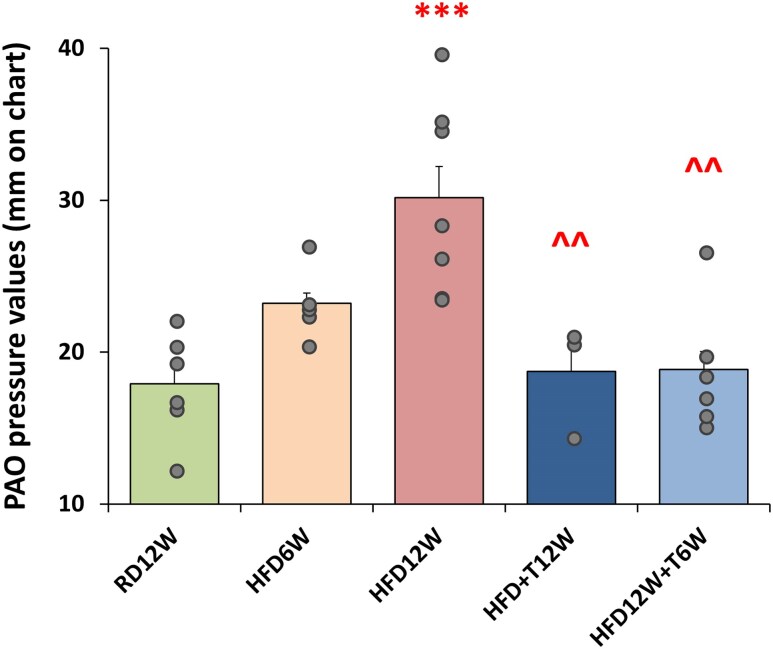
Effects of preventive in vivo T treatment on the pressure of airway opening in the experimental groups: RD12W (n = 7), HFD6W (n = 6), HFD12W (n = 7), HFD + T12W (n = 3), and HFD12W + T6W (n = 7). A small data subset (n = 3 from RD12W, HFD12W, and HFD + T12W groups) included here was reported in phase 1 of the project. Data are expressed as pressure values (in mm) recorded by the signal transducer and reported as mean ± SEM. Data were analyzed by 1-way parametric ANOVA test followed by post hoc Tukey honestly significant difference (HSD) test to evaluate differences between groups, with *P* < .05 considered as significant. ***P < .001 vs RD12W; ^^*P* < .01 vs HFD12W.

We next investigate respiratory function by physiological measurements of the PAO. PAO analyses (Fig. 4) revealed that the first signs of pulmonary functional alteration were already present at 6 weeks of HFD, with an overt alteration reported after 12 weeks of HFD, compared to RD12W (*P* < .001; Fig. 4). Remarkably, the 6-week T treatment completely normalized this functional parameter (*P* < .01 vs HFD12W; Fig. 4). Accordingly, also the 12-week T treatment showed a beneficial effect on PAO (*P* < .01 vs HFD12W; Fig. 4), albeit only tested on a limited number of animals in this group.

**Figure 5:**
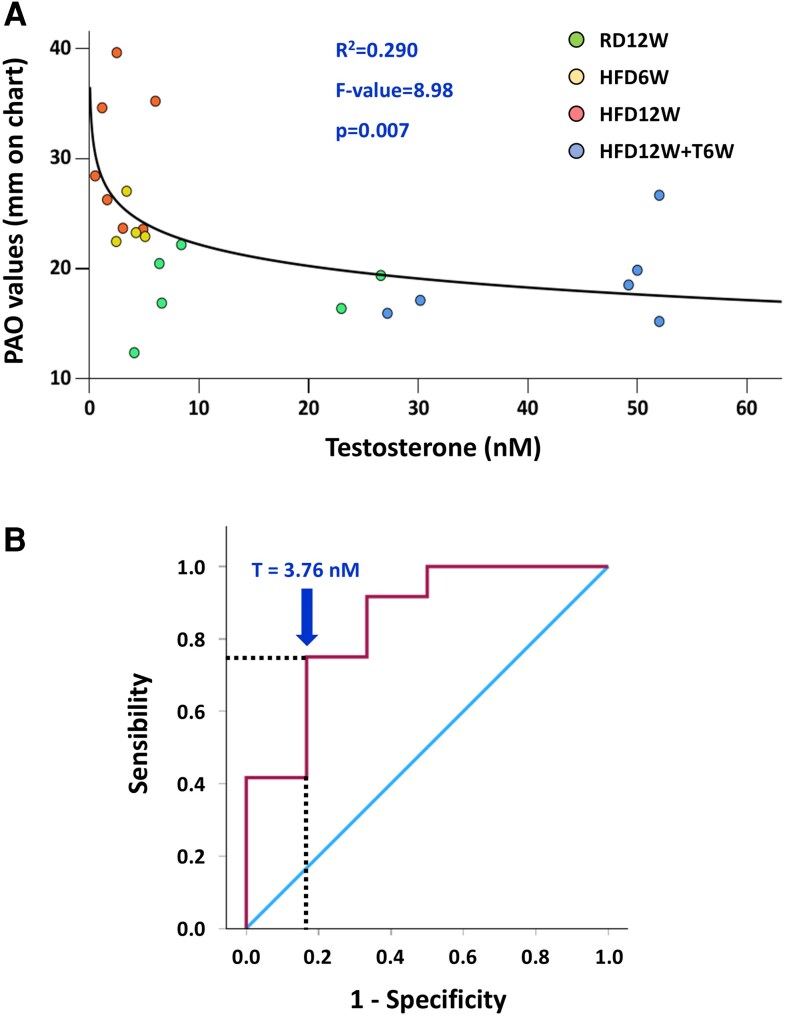
(A) The best-fitting model for the relationship between PAO and total T (logarithmic relationship). (B) The ROC curve analysis of these 2 parameters, with the dotted lines indicating the point with greater sensibility and specificity. Correlations were evaluated by Spearman bivariate correlation analyses and by multivariate analyses.

Moreover, Fig. 5A shows the negative relationship between T plasma levels and PAO in the different animal groups. The best fitting model is a logarithmic relationship (*R*^2^ = 0.290; *P* = .007; F value = 8.98, n = 23). Among MetS factors, PAO resulted significantly associated also with several metabolic parameters, including plasma lipids and glucose. The best fitting model resulted in a linear association with glycemia (*R*^2^ = 0.249, *P* = .011, F value = 7.64, n = 24), whereas a logarithmic relationship was observed for total cholesterol (*R*^2^ = 0.205, *P* = .023, F value = 5.92, n = 24) and triglycerides (*R*^2^ = 0.335, *P* = .003, F value = 11.08, n = 23). When the log-transformed (testosterone, triglycerides, cholesterol) or not (glycemia) aforementioned parameters were introduced as covariates in a multivariate analysis, only the relationship between PAO and T retained significance (β = −0.425, *P* = 009).

In RD12W, mean (±SD) PAO level was 17.92 ± 3.5 mm (Fig. 4). We defined normal PAO as the mean ± 2 SD of RD12W levels (ie, 25 mm). In T-untreated animals (RD12W; HFD6W and HFD12W), we used this parameter to evaluate the critical T level associated with a pathological PAO (<25 mm) through receiver operating curve analysis. The results are reported in Fig. 5B, and a T value below 3.76 nM was able to identify an abnormal PAO (<25 mm) with a sensibility of 0.75, a specificity of 0.83 and an accuracy of 0.847 (*P* = .001).

#### Phase 2—immunohistochemical analyses of lung tissue in HFD for 6 or 12 weeks with or without 6 weeks of T administration

**Figure 6:**
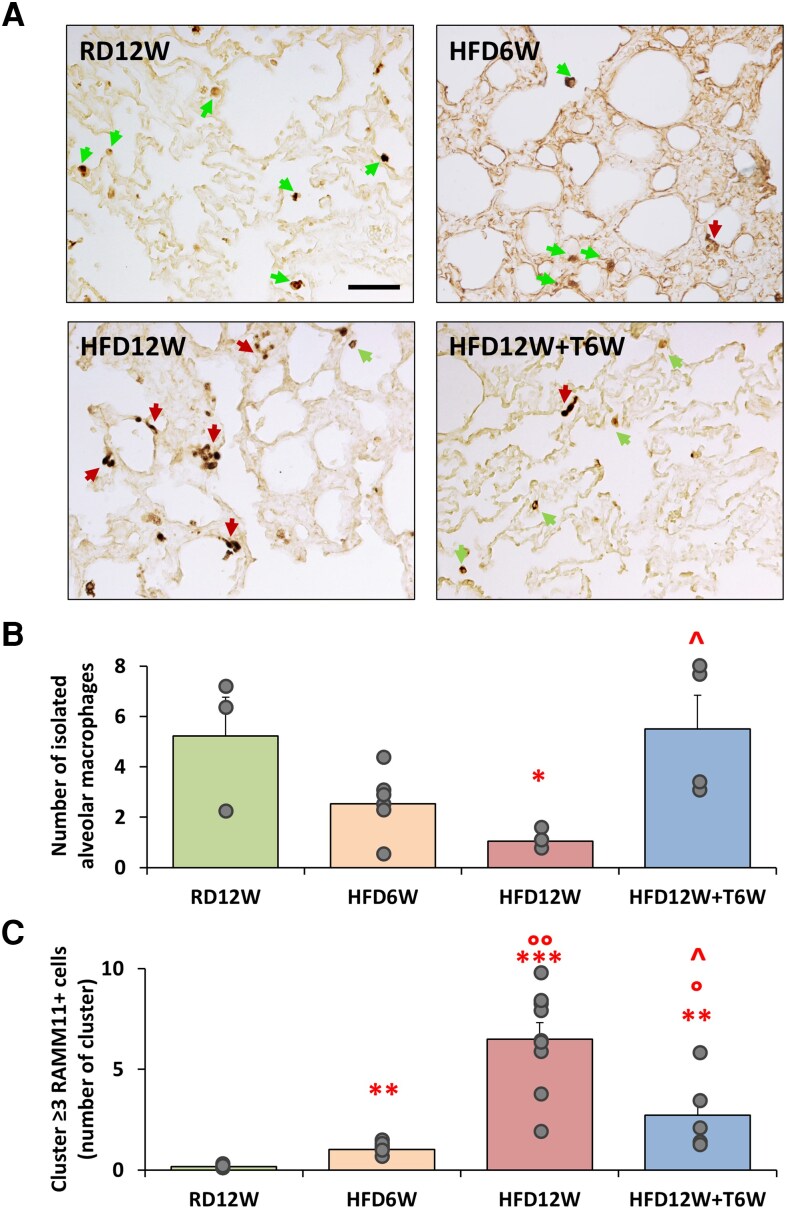
RAM11 immunostaining and molecular analyses. (A) Representative images of RAM11 immunostaining in lung sections for all experimental groups. Green arrows indicate isolated alveolar macrophages, whereas red arrows highlight macrophage clusters (3 or more cells). Scale bar = 50 µM. (B-C) Quantification of the isolated resident macrophages (n ≥ 3 per group) and the monocyte-derived activated macrophage clusters (n ≥ 5 per group), respectively. To evaluate differences between groups, with *P* < .05 considered as significant, data were analyzed by either 1-way parametric ANOVA test followed by post hoc Tukey honestly significant difference (HSD) test (for normally distributed parameters) or 1-way nonparametric Kruskal-Wallis ANOVA followed by post hoc Mann-Whitney analysis (for not normally distributed parameters). *P < .05, **P < .01, ***P < .001 vs RD12W; °*P* < .05, °°*P* < .01 vs HFD6W; ^*P* < .05 vs HFD12W.

As shown in the different panels of Fig. 6, RAM11 immunostaining revealed that, compared to RD12W (panel A), the isolated alveolar macrophages are depleted in both HFD groups (panels A-C); likewise, the pro-inflammatory monocyte derived macrophage clusters are significantly increased by both HFD regimes (panels A-C). Six-week T treatment (panel A) was able to significantly improve both parameters, compared to HFD12W, in particular bringing the number of isolated alveolar macrophages back to RD levels, as reported in the quantification bar graphs (panels B and C).

**Figure 7:**
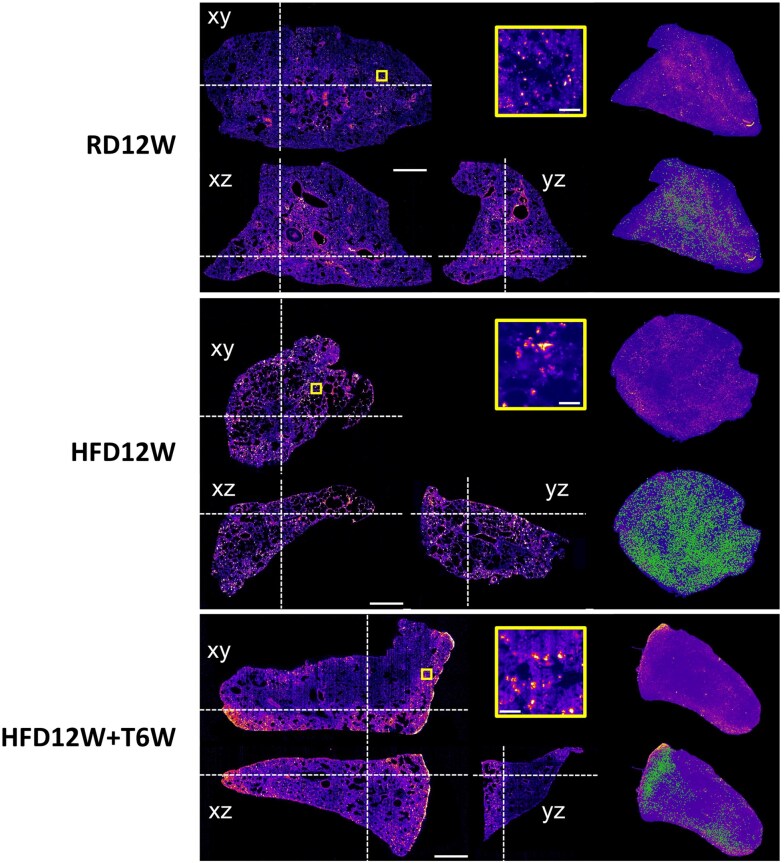
Volumetric analysis of lung samples. Orthogonal views and 3D rendering of rabbit lung samples stained against RAM11. Dashed white lines identify the orthogonal slices shown in the same figure. Yellow boxes show insets at full resolution, demonstrating the capability to identify isolated cells or cell clusters. In the renderings, green dots identify cell clusters, defined as segmented areas larger than 500 voxels. Scale bars: 1 mm (orthogonal views), 100 μm (insets).

In addition, using the tissue clearing technique coupled with 3D DISCO microscopy, we further documented the extent of the inflammatory status by assessing the spatial distribution of RAM11-positive cells within the whole 3D lung volume of each group (Fig. 7). The volumetric analysis showed that in lung samples from HFD rabbits macrophage clusters, as detected by voxel > 500, were strongly evident and widely distributed within the entire tissue volume, in comparison to RD sample (Fig. 7). The lung sample from T-treated HFD rabbit exhibited a diminished macrophage clusterization, confined to some areas of the 3D image.

To further explore the HFD effects on lung function and potential mechanisms responsible for the effect of T treatment for 6 weeks, we analyzed lung morphology and structure by specific histological staining.

**Figure 8:**
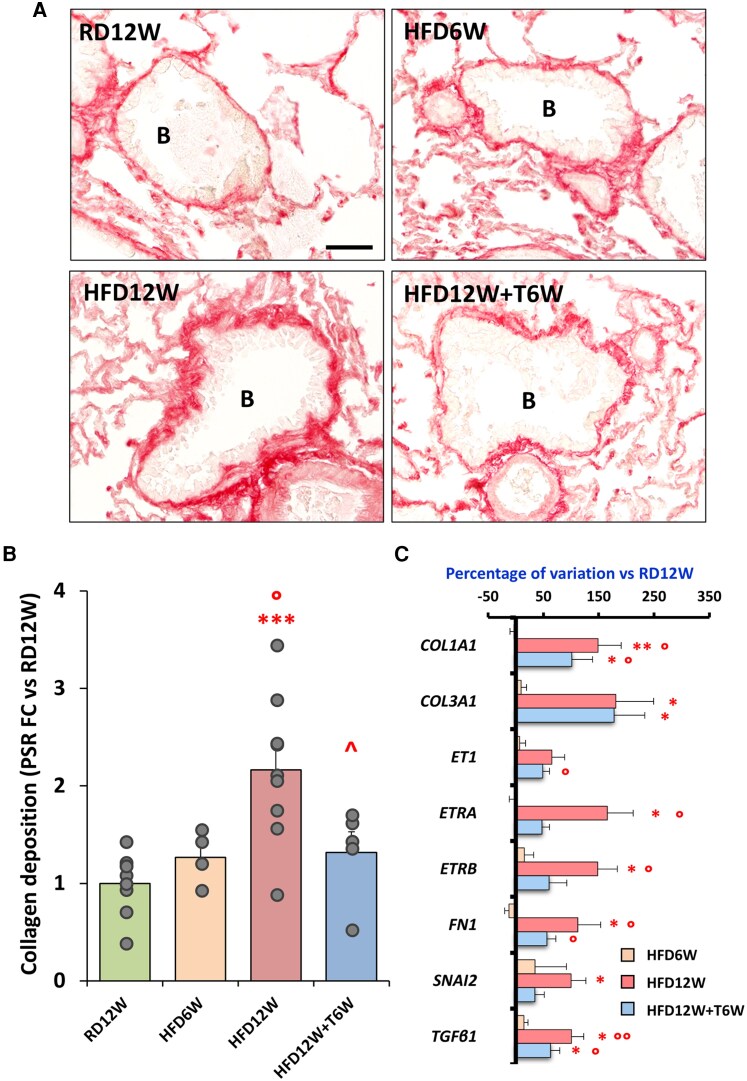
Picrosirius red analyses of peribronchiolar fibrosis. (A) Representative images of all experimental groups showing collagen deposition in the peribronchiolar area. (B) Bronchiole. Scale bar = 50 µM. (B) Percentage of collagen deposition in the bronchiolar perimeter relative to the total bronchiolar area (n ≥ 4 per group). Data are expressed as fold change vs RD12W and reported as mean ± SEM. (C) Semiquantitative lung mRNA expression of pro-fibrotic markers in the RD12W (n = 10), HFD16W (n = 6), HFD12W (n = 12), and HFD + T12W (n = 7) experimental groups. Results are expressed as mean ± SEM and reported as fold-change vs RD12W. To evaluate differences between groups, with *P* < .05 considered as significant, all biomarkers were analyzed by: (A) for normally distributed parameters either 1-way parametric ANOVA test followed by post hoc Tukey honestly significant difference (HSD) test (B) or parametric Welch ANOVA test followed by post hoc Games-Howell test (C), according to Levene variance test; (B) for not normally distributed parameters 1-way nonparametric Kruskal-Wallis ANOVA followed by post hoc Mann-Whitney analysis (C). *P < .05, **P < .01, ***P < .001 vs RD12W; °*P* < .05, °°*P* < .01 vs HFD6W; ^*P* < .05 vs HFD12W.

**Figure 9:**
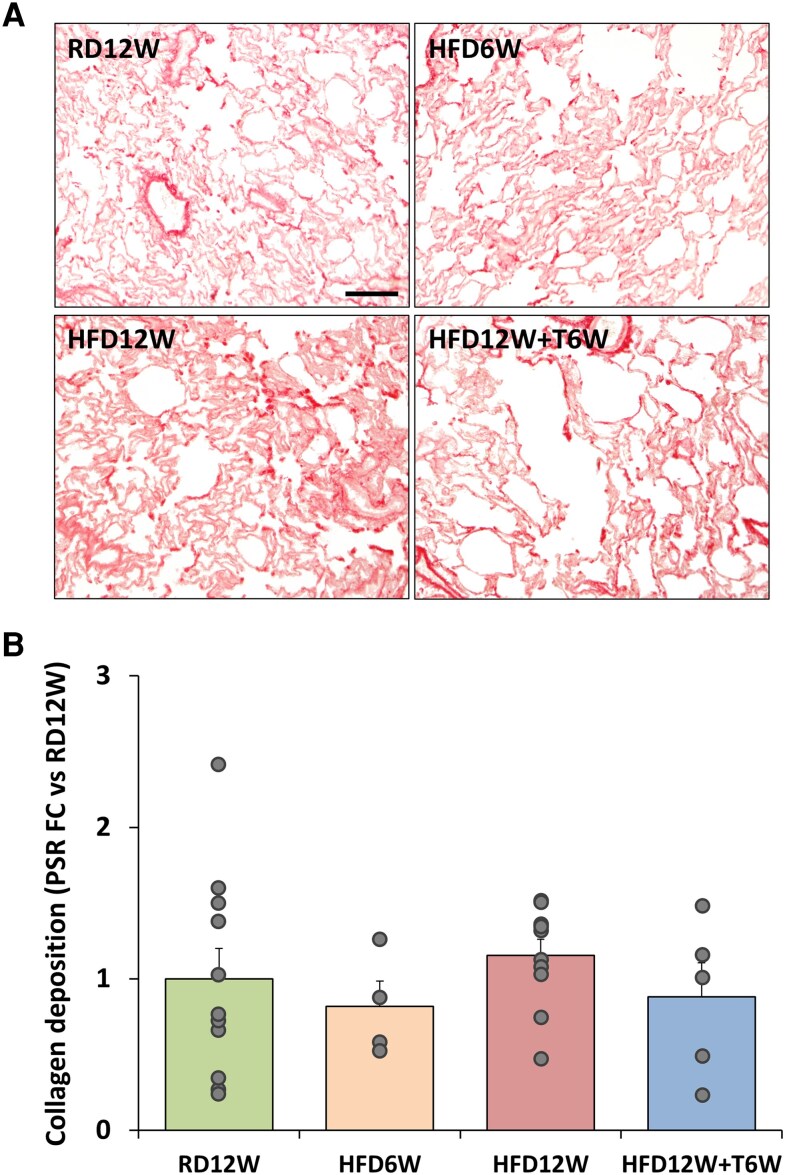
Picrosirius red analyses of parenchymal fibrosis. (A) Representative images of fibrosis distribution in lung parenchyma for all experimental groups. Scale bar = 50 µM. (B) Percentage of fibrosis area in rabbit lung sections (n ≥ 6 per group). Data are expressed as fold-change vs RD12W and reported as mean ± SEM. Data were analyzed by 1-way parametric ANOVA test, with *P* < .05 considered as significant (ANOVA, *P* = .638).

Collagen deposition, evaluated by picrosirius red staining, evidenced the presence of peribronchiolar fibrosis in lung sections from the 12-week HFD group, but not after 6-week HFD (Fig. 8A). This was significantly counteracted by 6-week T treatment (*P* < .01 vs HFD12W), as also demonstrated by the quantification of the collagen deposition area (Fig. 8, panel B). As reported in Fig. 8C, HFD6W partially induced, and HFD12W significantly increased, the mRNA expression of remodeling markers. The 6-week T treatment promoted a downregulation of the mRNA expression of several of the genes involved in pro-fibrotic processes. No changes among groups were detected in terms of parenchymal collagen deposition (Fig. 9A, B).

#### Phase 2—molecular analyses of lung tissue in HFD for 6 or 12 weeks with or without 6 weeks of T administration

**Figure 10:**
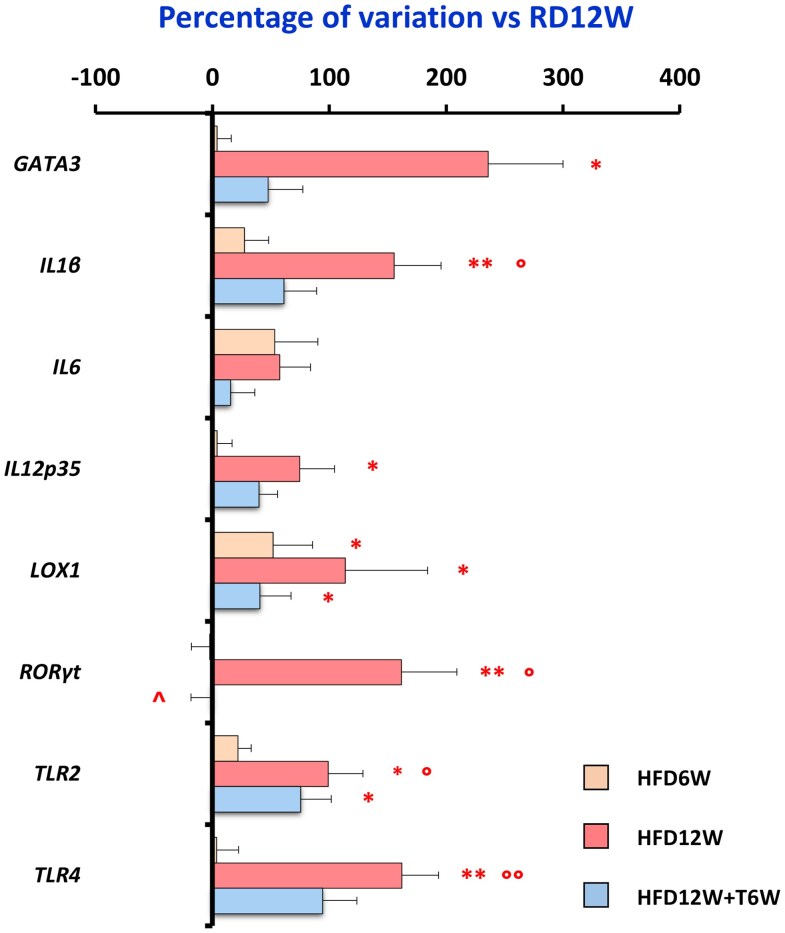
Effects of preventive in vivo T treatment on pro-fibrotic and pro-inflammatory molecular targets in the HFD6W (n = 6), HFD12W (n = 12), and HFD12W + T6W (n = 7) experimental groups. Results are expressed as percentage of variation vs RD12W (n = 10) and reported as mean ± SEM. To evaluate differences between groups, with *P* < .05 considered as significant, all biomarkers were analyzed by either 1-way parametric ANOVA test followed by post hoc Tukey honestly significant difference (HSD) test (for normally distributed parameters) or 1-way nonparametric Kruskal-Wallis ANOVA followed by post hoc Mann-Whitney analysis (for not normally distributed parameters). *P < .05, **P < .01 vs RD12W; °*P* < .05, °°*P* < .01 vs HFD6W; ^*P* < .05 vs HFD12W.

In Fig. 10 we reported the mRNA expression of inflammatory genes after 6 and 12 weeks of HFD, and the effect of T treatment for the last 6 weeks. As shown, 6 weeks of HFD numerically increased several inflammatory genes, without reaching statistical significance for the majority of them. As additionally reported in Fig. 10, 12 weeks of HFD significantly increased several proinflammatory genes. T administration 6 weeks before the sacrifice was able to counteract that increase, showing level of inflammatory genes similar to RD12W, albeit still slightly increased.

Crucially, 6-week T treatment reduced significantly, compared to HFD12W (*P* < .05), the mRNA expression of *RORγt*, a molecule of paramount importance in the recruiting of macrophages.

**Figure 11:**
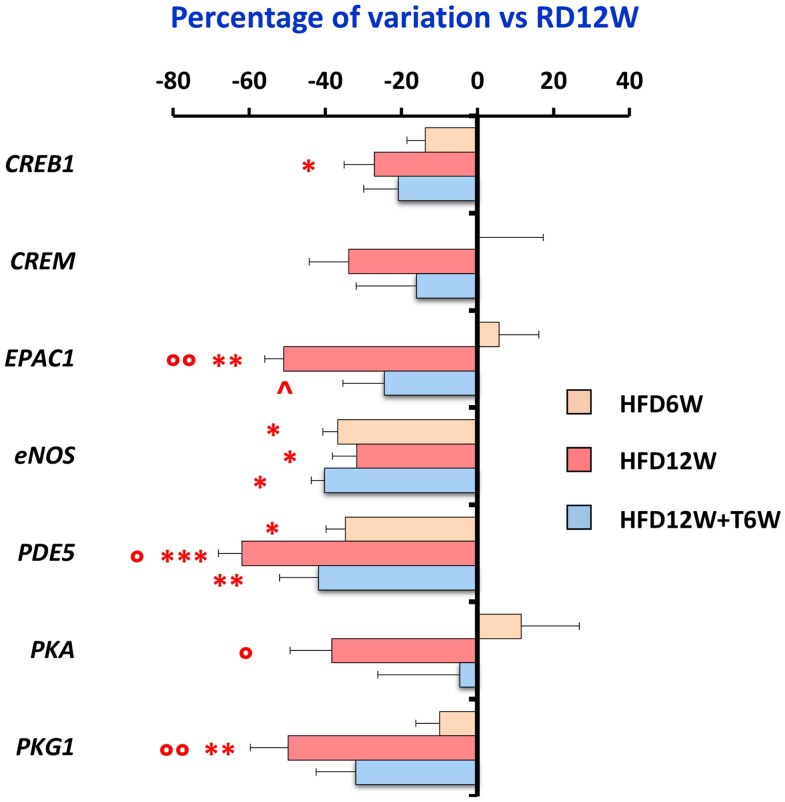
Effects of preventive in vivo testosterone treatment on NO/cGMP and cAMP signaling molecular targets in the HFD6W (n = 6), HFD12W (n = 12), and HFD12W + T6W (n = 7) experimental groups. Results are expressed as percentage of variation vs RD12W (n = 10) and reported as mean ± SEM. To evaluate differences between groups, with *P* < .05 considered as significant, all biomarkers were analyzed by either 1-way parametric ANOVA test followed by post hoc Tukey honestly significant difference (HSD) test (for normally distributed parameters) or 1-way nonparametric Kruskal-Wallis ANOVA followed by post hoc Mann-Whitney analysis (for not normally distributed parameters). *P < .05, **P < .01, ***P < .001 vs RD12W; °*P* < .05, °°*P* < .01 vs HFD6W; ^*P* < .05 vs HFD12W.

We thereafter investigated the mRNA expression of genes involved in pathways linked to cGMP and cAMP signaling. Figure 11 shows that the mRNA expression of cGMP related genes was significantly decreased by HFD, particularly at week 12. A significant decrease was also observed for cAMP signaling related genes. The 6-week dosing of T did not appear to significantly affect cGMP signaling, whereas a significant (*EPAC1*) or evident (*CREB1*, *CREM*, *PKA*, and *PKG1*) recovery for mRNA expression was observed, indicating that the cAMP pathway might be potentially involved in the T effect on the lung.

## Discussion

In this study, we demonstrated that an experimentally induced metabolic syndrome in rabbits induces relevant morphological and pulmonary functional alterations by promoting inflammatory and pro-fibrotic processes. Our data thus highlight that a MetS-associated pulmonary alteration might be considered as a new aspect of the clinical MetS scenario, that also includes male hypogonadism (4).

Interestingly, T treatment was able to improve lung inflammatory and fibrotic damage. However, it remains to be established whether the beneficial effect of T on pulmonary function is mediated by a general improvement of the MetS condition or is a direct effect of T on the lung tissue. Indeed, we found a relatively high expression of *AR* in lung, as well as a significant relationship between a lung functional parameter (pressure at airway opening, PAO) and T level.

In animal models, fibrotic lung conditions, including idiopathic pulmonary and bleomycin-induced fibrosis, are characterized by a marked shift in alveolar macrophage populations with a loss of tissue-resident alveolar macrophages (TR-AMs) and the influx, differentiation, and expansion of monocyte-derived alveolar macrophages (Mo-AMs) (48, 49). The shift from a relatively homogeneous population of TR-AMs to a heterogeneous array of macrophage subsets is demonstrated by the emergence of distinct macrophage subsets in fibrotic lungs. These subsets follow a temporal trajectory from monocytes to active macrophages (50, 51).

Under normal conditions, TR-AMs contribute to lung homeostasis by regulating immune responses, clearing surfactant, and facilitating tissue repair. However, fibrotic insults lead to selective depletion of TR-AMs, likely because of mechanisms such as oxidative stress, apoptosis, or disrupted niche signaling (48). In contrast, alveolar injury triggers the release of chemokines, which recruit monocytes from the bone marrow. These monocytes infiltrate the lung and differentiate into Mo-AMs, which adopt a transcriptional and phenotypic profile distinct from that of TR-AMs (48, 52). Mo-AMs persist long term in the fibrotic lung and express elevated levels of profibrotic mediators involved in fibroblast activation, extracellular matrix remodeling, and tissue stiffening (49, 52).

Our data show that MetS induced TR-AM clustering and activation, along with an increase of pro-inflammatory and pro-fibrotic markers, supporting the concept that this tissue-resident macrophage population could also exert a critical role for MetS-related lung disease. Indeed, a specific deletion of population of macrophages in the brain, intestine, and adipose tissue was demonstrated to be protective against obesity and MetS (53). From a mechanistic point of view, all these changes could be the consequence of circulating pro-inflammatory insults deriving from the HFD-induced metabolic perturbation, such as those linked to dyslipidemia and increased circulating fatty acids. For instance, it has been demonstrated in vivo that HFD, and in detail increased dietary LCFAs (such as stearic acid) was able to alter the resident macrophage phenotype and function, thus promoting lung response to pro-inflammatory insults (54).

This finding is consistent with the significant increase in stearic and palmitic acids plasma concentration observed in HFD rabbits. Accordingly, palmitic acid is one of the stronger activators of the TLR4 signaling pathway (55). In lung from HFD animals, we found a sharp increase of several inflammatory markers such as *TLR2* and *TLR4*, which are known to be specifically expressed in lung alveolar macrophages (56), as well as *RORyt* and *GATA3*, which are specific markers for T helper 17 (Th17) and T helper 2 (Th2), respectively. A complex interplay between other Th17 and Th2 lymphocytes with fibroblasts is then responsible for lung fibrosis with an excessive extracellular matrix deposition, leading to obliteration of the original tissue architecture and function (57).

Accordingly, HFD demonstrated an additional and significant increase of lung fibrosis as assessed either by using immunohistochemical staining or by mRNA expression of several genes related to pro-fibrotic and tissue remodeling process such as *ET1* (with its 2 cognate receptors, *ETRA* and *ETRB*) and *TGFβ* (with its transcriptional factor, *SNAI1*). In detail, IL1β and TGFβ along with SNAI1 have been also implicated in inflammation-driven epithelial-mesenchymal transition, which is the phenotypic switching of alveolar epithelial type II cells to fibroblast-like cells leading to tissue fibrosis (58). Indeed, in lung from HFD animals we also found a significant increase of extracellular matrix deposition markers, namely *COL1A1*, *COL3A1* and *FN1*. Interestingly, we also evidenced a significant increase of *LOX1*, the receptor for oxidized low-density lipoprotein, which increased also in lipopolysaccharide-induced lung injury (59). LOX1 blockade has been described to prevent acute lung inflammation and injury (60). Therefore, these data pointed toward a potential pathogenic role of metabolic factors in first triggering the inflammatory process then leading to fibrotic remodeling in the lung. Indeed, among all the inflammatory markers, *LOX1* was the one that demonstrated the most relevant increase already at the earlier stage (6 weeks) of HFD-induced lung injury.

In addition, HFD significantly decreased the mRNA expression of several genes related to cGMP and cAMP signaling pathways. Interestingly, cAMP recently garnered clinical attention, because of its ability to elicit antifibrotic effects and spontaneous fibrosis resolution, either in preclinical or clinical model (61). Noteworthy is that the cAMP relevance in preventing progressive scarring and restoring lung homeostasis has been recently demonstrated in a phase 3 trial where nerandomilast (a PDE4B inhibitor that increases intracellular cAMP) was able to slow the progression of idiopathic pulmonary fibrosis (62).

Functionally, we observed a severe PAO increase in HFD animals that mirrors an overt and clinically evident lung injury induced by MetS. As previously reported in MetS condition in the clinical setting (12), HFD animals showed the onset of an overt condition of hypogonadism not only after 12 weeks (21, 26-29), but also after 6 weeks of HFD administration. This earlier time point (6 weeks) was also characterized by the onset of several MetS alterations, so we tested the effects of 2 distinct protocols of testosterone administration: 1 preventing (starting T administration from time 0 along with HFD for 12 weeks) and 1 therapeutic (starting T administration after 6 weeks of HFD for other 6 weeks to the 12th week). In both protocols, we were able to demonstrate a clear-cut decline of HFD-induced alterations of pro-inflammatory and fibrotic processes.

Above all, we observed that T treatment reduced inflammatory genes, such as those related to macrophages (*TLR2* and *TLR4*) as well as Th2 (*GATA3*) and Th17 (*RORyt*) immune response, together with several profibrotic markers (*COL1A1*, *COL3A1*, *ET1*, *ETRA*, *ETRB*, *TGFβ*, *SNAI*). This mRNA expression improvement was observed not only in the preventive but also, and more notably, in the therapeutic protocol of T administration by which we additionally demonstrated a lung morphological and functional improvement. In immunohistochemical analysis 6 weeks administration of T was indeed able to counteract HFD-induced macrophages cluster activation and fibrosis. The lack of overt parenchymal lung fibrosis in all HFD groups, with the presence of peribronchiolar collagen deposition in the 12-week HFD and not 6-week HFD animals, further supports the mechanistic hypothesis of MetS-related inflammatory insults as first tissue damage, starting at peribronchiolar level, further highlighting the anti-inflammatory nature of protective role of T treatment able to counteract the progression towards pro-fibrotic processes. Noteworthy, in the functional study T6W demonstrated a significant PAO decrease.

We report that T could exert a positive effect on pulmonary function, and that lung should be considered as a new and important target tissue for androgen activity. We found that *AR* is expressed in lung epithelial cells at relatively high level, although lower than in prostate, which is a typical androgen-dependent gland. In the multivariate analysis, we found that among all the MetS-associated factors, T levels were independently associated with lung functioning (PAO), in an exponential manner. A T value below 3.76 nM was able to identify an abnormal PAO with highly significant sensibility, specificity, and accuracy.

A limitation of the study is that, for the RD groups, only biochemical and metabolic parameters were assessed at the 6-week time point in the RD12W control group. This decision was made in adherence to the 3Rs principles of animal research to minimize animal use and avoid unnecessary culling. Consequently, functional, molecular, and histological comparisons at 6 weeks under a normal diet were not possible. Because RD animals did not develop any lung dysfunction after 12 weeks, HFD6W animals were compared to RD12W controls rather than age-matched RD6W animals, which should be considered when interpreting early HFD-induced functional and molecular effects. The present study shows some other limitations, considering that the obtained data cannot clearly establish whether the T-induced lung improvements here described are a direct consequence of pulmonary AR stimulation or just the result of a decreased MetS severity. In addition, generalization from a preclinical model to the human condition needs further investigations. Nonetheless, from a clinical standpoint, this underscores the need of a thorough assessment and management of metabolic and hormonal disorders in individuals with, or at risk for, respiratory disease. Further research is needed to determine whether interventions like testosterone replacement therapy and metabolic optimization can directly decrease respiratory morbidity and mortality. It should also be noted that circulating estradiol levels were not measured in the present study. However, our previous work showed that a HFD doubled estradiol levels compared to RD (*P* < .001) (31, 63). Testosterone treatment, in contrast, restored estradiol to baseline levels, with estradiol showing a negative correlation with testosterone and a positive correlation with the number of metabolic syndrome components, in line with the minimal aromatase activity observed in rabbits (31).

Collectively, these findings highlight in a preclinical model the emergence of a MetS-associated lung disorder. This condition is characterized by an inflammatory and profibrotic process that impairs pulmonary function. Although metabolic alterations and a concurrent hypogonadal state may synergistically contribute to these observed lung changes, a more pronounced and independent role of low T levels is also recognized. This is supported by evidence that T administration, in both preventive and therapeutic contexts, effectively counteracts the majority of HFD-induced pulmonary alterations.

Our study, by analyzing the functional, fibrotic, and inflammatory patterns in the lungs of rabbits with MetS and assessing the impact of T treatment on these pulmonary changes, provides critical insights into the underlying mechanisms and potential therapeutic targets related to metabolic dysregulation. This evolving understanding presents novel opportunities for precision therapies. In summary, this research results support the development of personalized, targeted prevention and intervention strategies—such as tailored T treatment protocols—for patients with MetS, hypogonadism, and related lung conditions. In the long term, identifying effective therapies in this setting could significantly reduce the societal burden of the disease by lowering health care costs and improving both the quality of life and life expectancy of affected patients.

## Data Availability

Original data generated and analyzed during this study are included in this published article. Further inquiries can be addressed to the corresponding author.

## Abbreviations

AR: androgen receptor
CV: cardiovascular
HFD: high-fat diet
LCFA: long-chain fatty acid
LOD: lower limit of detection
MAP: mean arterial blood pressure
MeTs: metabolic syndrome
Mo-AM: monocyte-derived alveolar macrophage
OGTT: oral glucose tolerance test
PAO: airway opening pressure
RD: regular diet
T: testosterone
TR-AM: tissue-resident alveolar macrophage
VAT: visceral adipose tissue
